# The British Journals: Anatomy and Physiology

**Published:** 1840-01

**Authors:** 


### III. THE BRITISH JOURNALS.

(FOR THE QUARTER ENDING NOVEMBER 30, 1839.)

### ANATOMY AND PHYSIOLOGY.

*On the Influence of Madder on the Bones of growing Animals.* By JAMES PAGET, Esq., Demonstrator of Morbid Anatomy, and Curator of the Museum at St. Bartholomew's Hospital.

[THIS paper is valuable, not so much for supplying fresh materials to physiology, as for enabling us (a thing of almost equal importance) to make a proper use of former materials. We can only find room for the introductory and the concluding paragraphs.]

The value of experiments made for the purpose of observing the growth of bones, and more remotely the general process of nutrition, by feeding growing animals on madder, has been, at different times, very differently estimated. They have been supposed by some to afford the only certain proof of a constant change of particles in nutrition, while by others they have been considered so vague and uncertain as to admit of no profitable application; and it would be easy to quote from the highest physiological authorities expressions of such doubt, with respect to the conclusions to be drawn from them, as would at once justify any attempt to explain their difficulties and apparent contradictions.

The general result of these experiments, then, is that which Du Hamel stated—that the increase in the length of bones is effected by the growth of those parts which are not yet hardened. Ossification commencing in the middle of the shaft, and proceeding rapidly towards its extremities, it is only in very young animals that any increase in the middle can be perceived; the later increase of length is due to the addition of osseous matter to the parts not yet hardened; and at the latest periods of growth is effected entirely by the ossification and reproduction of the intermediate cartilage between the shaft and the epiphysis.

In their applications to the explanation of the growth of bones, experiments with madder are thus proved to be conclusive: with a knowledge of the principles on which they depend, and with a careful avoidance of some sources of fallacy that have been pointed out, they may be employed with entire confidence; they afford to the observer the means, as it were, of branding with his own mark every particle of phosphate of lime that is deposited during a given time.

Their applications to the general doctrine of nutrition are less certain: for, in the first place, it is not clear that the laws which govern the production and maintenance of the earthy and inorganic matter of bones are applicable to the nutrition of soft parts; and if they be, the result of the madder experiments is at present, in this point of view, entirely negative, if not opposed to the idea of a constant mutation of particles. Seeing that the blood containing madder has no evident influence on the phosphate of lime already deposited in the bones, and that, on the other hand, blood not containing madder does not possess any chemical power to abstract the colour from the coloured phosphate of lime, it would appear as if there were no influence exerted during the state of health between the contents of the capillary system and the earthy matter already existing in the bones. And as, moreover, the bones of old animals are coloured by madder only to that degree which appears explicable by their increase of density, it would seem as if, in the state of health, or in what I would call the state of *nutritive equilibrium*, the earthy matter of bones undergoes no change.

*London Medical Gazette. Nov.* 15, 1839.

